# P289: Prevalence and risk factors of hepatitis c virus infection in chronic hemodialysis patients at the university teaching hospital of point g, Bamako, Mali

**DOI:** 10.1186/2047-2994-2-S1-P289

**Published:** 2013-06-20

**Authors:** M Baby, S Fongoro, MK Konaté, A Diarra, B Kouriba, MK Maïga

**Affiliations:** 1 National Center for Blood Transfusion, University Hospital of Point G, Bamako, Mali; 2 Department of Nephrology, University Hospital of Point G, Bamako, Mali

## Objectives

The objective of this prospective study conducted in November 2008, was to determine the prevalence and the factors associated with Hepatitis C Virus (HCV) infection in chronic hemodialysis patients.

## Methods

The study was carried out in the hemodialysis unit of the university teaching hospital of Point G. Serum samples were tested for anti-HCV antibody, anti-HIV antibody and HBs Ag using enzyme immunoassay methods (ELISA) at the laboratory of immunology of the National Blood Transfusion Service of Bamako. The following parameters were assessed: initial nephropathy, duration of the dialysis, history of blood transfusion, number of blood units transfused since the beginning of the dialysis, history of nosocomial exposure.

## Results

A total of 66 patients were enrolled. The mean age of the patients was 42,27±14, 8 years, with a male to female sex-ratio of 1,44. Anti-HCV antibodies were found in 13 chronic hemodialysis patients, leading to a prevalence of 19,7%. A significant association was found between the bearing of HCV and the duration of the dialysis.

## Conclusion

These results indicate that hepatitis C is frequent in the chronic hemodialysis patients of the university teaching hospital of Point G, and that the duration of dialysis constitutes the main factor associated with the contamination by the HCV.

## Competing interests

None declared

